# Multifaceted Value Profiles of Forest Owner Categories in South Sweden: The River Helge å Catchment as a Case Study

**DOI:** 10.1007/s13280-012-0374-2

**Published:** 2013-03-10

**Authors:** Gustav Richnau, Per Angelstam, Sviataslau Valasiuk, Lyudmyla Zahvoyska, Robert Axelsson, Marine Elbakidze, Joshua Farley, Ingemar Jönsson, Ihor Soloviy

**Affiliations:** 1 Landscape Management, Design and Construction, Swedish University of Agricultural Sciences, 230 53 Alnarp, Sweden; 2 Faculty of Forest Sciences, School for Forest Management, Swedish University of Agricultural Sciences, PO Box 43, 730 91 Skinnskatteberg, Sweden; 3 Faculty of Economic Sciences, University of Warsaw, ul. Długa 44/50, 00-241 Warszawa, Poland; 4 Institute of Ecological Economics, Ukrainian National Forestry University, 103 Gen. Chuprynky, Lviv, 79057 Ukraine; 5 Faculty of Forest Sciences, School for Forest Management, Swedish University of Agricultural Sciences, PO Box 43, 739 21 Skinnskatteberg, Sweden; 6 Department of Community Development and Applied Economics, UVM College of Agriculture and Life Sciences, Morrill Hall, Burlington, VT 05405 USA; 7 School of Education and Environment, Kristianstad University, 291 88 Kristianstad, Sweden

**Keywords:** Economic sustainability, Kristianstad Vattenrike Biosphere Reserve, Forest management, Landscape governance

## Abstract

Forest landscapes provide benefits from a wide range of goods, function and intangible values. But what are different forest owner categories’ profiles of economic use and non-use values? This study focuses on the complex forest ownership pattern of the River Helge å catchment including the Kristianstad Vattenrike Biosphere Reserve in southern Sweden. We made 89 telephone interviews with informants representing the four main forest owner categories. Our mapping included consumptive and non-consumptive direct use values, indirect use values, and non-use values such as natural and cultural heritage. While the value profiles of non-industrial forest land owners and municipalities included all value categories, the forest companies focused on wood production, and the Swedish Environmental Protection Agency on nature protection. We discuss the challenges of communicating different forest owners’ economic value profiles among stakeholders, the need for a broader suite of forest management systems, and fora for collaborative planning.

**Electronic supplementary material:**

The online version of this article (doi:10.1007/s13280-012-0374-2) contains supplementary material, which is available to authorized users.

## Introduction

The success of implementing any policy decision aimed at changing human behavioral patterns depends on our knowledge of the drivers underlying the perceptions of a landscape’s actors and stakeholders towards forest ecosystems and their services. A key challenge is to incorporate multifaceted tangible and intangible landscape values into governance and management processes (Szaro et al. 2005; Lindström et al. 2006; Sturtevant et al. 2007; Kumar 2010; Axelsson et al. 2013a). For example, there are significant gaps between the way we describe and monitor forest landscapes in practice (e.g., focus on wood and biomass at the stand scale) and what ought to be the case if based on the current definition of policies on sustainable natural resource use (e.g., also including non-timber forest products as well as ecological, social, and cultural dimensions at multiple scales) (Innes and Hoen 2005).

Contemporary mainstream economics is a discipline that deals with decision-making when natural resources are scarce. It applies an anthropocentric utilitarian approach (Merlo and Croitoru 2005), where willingness to pay is treated as equivalent to utility provided. The economic value of a good is therefore determined by preferences weighted by purchasing power. Consumers can express their choice among different bundles of market goods and services in monetary terms, and the resulting values can be incorporated into standard cost-benefit analyses when necessary (e.g., when governments must make investment decisions) (WCPA 1998). However, the problem with many landscape goods and services is the lack of appropriate markets for them, or noticeable distortions on the existing markets (Barbier et al. 1997). In the case of landscape goods that qualify as public goods, markets either fail to set a price or else the equilibrium price is not optimal in the Pareto sense (Samuelson 1954). In general, ecosystem goods (e.g., timber and fish) can readily be converted into market goods. In contrast, many of the services generated by intact ecosystems are public goods, which means that the very existence of regular institutionalized markets for them is not feasible and there is in principle no market price. The result is permanent bias of decision-making mechanisms favoring ecosystem conversion over conservation, regardless of which option maximizes benefits to society as a whole (Farley 2010).

Extending the scope of economics beyond marketed goods, services and values relies on relevant instruments and mechanisms. Much economic literature focuses on theories of non-market valuation, and there exists a variety of valuation techniques with various strengths, weaknesses, and applicability (Krutilla 1967; Arrow et al. 1993; Hausman 1993; Carson et al. 2001; Bateman and Willis 2002; Carson and Hanemann 2005). These methods can be divided into two major groups, namely methods based on revealed preferences and based on stated preferences, respectively. The first group is based on preferences revealed by economic agents in actually existing markets while the second group relies on people’s preferences registered in hypothetical choice situation rather than on signals obtained from actual market transactions. In many cases the use of stated preferences is the only approach consistent with economic theory.

An extensive body of scientific literature on economic valuation of environmental assets in general and of forest ones in particular demonstrates success of environmental economists in encompassing measurement of environmental values, but also methodological limitations of the different valuation techniques (Arrow et al. 1993; Bateman and Willis 2002; Kant 2003) as well as of econometric models traditionally applied for the monetary appraisal (Meadows 2008). Ecological economists, however, take a different approach to valuation of natural resources and ecological functions. It is based on the precautionary principle and argues that the stock of natural resources and ecological functions are irreplaceable since their destruction could be irreversible, thus focusing on strong sustainability sensu Neumayer (2010). Kant and Lee (2004) argued that a continuum of values is closer to the concept of “social states” (de Borda 1781) rather than to the concept of “market prices”, especially in the context of non-use values. Therefore social choice theory and techniques based on this theory may be used to elicit and identify owners’ values, preferences, and attitudes associated with forest, forest values, and forest management practices (Kearney and Kaplan 1997; Nijnik and Mather 2008; Nijnik et al. 2009).

The wide range of benefits that forest and woodland landscapes provide implies a major analytic and methodological challenge. As utility in general is considered a source of economic value, forest landscapes provide people with various sorts of utility which are heterogeneous in their origin and features. Three main value components correspond to the various types of utility, namely use value, option value, and non-use value (Weisbrod 1964; Krutilla 1967). People may derive utility either from use (direct or indirect) of the forest landscape or a particular component, or from temporarily setting it aside for future use, or from leaving it intact for different reasons. Since related management strategies may contradict each other, decision-makers are forced to make trade-offs among different values, thus optimizing preferences and deriving maximal utility for individual stakeholders or society.

The Kristianstad Vattenrike Biosphere Reserve (KVBR) in southernmost Sweden (Hahn et al. 2006; Olsson et al. 2007) was one of the model sites in the Millennium Ecosystem Assessment. KVBR is a semi-urban area with high biological and cultural values located in the lower parts of the River Helge å catchment. The primary focus is on the wetlands area along the lowland part of Helge å river that was designated a Ramsar wetland in 1975. KVBR provides an attempt to create a collaborative governance system that includes a wide range of stakeholders (Olsson et al. 2004, 2007; Hahn et al. 2006). However, several important challenges require that a catchment approach is encouraged. For example, humic acid concentrations in the water have increased during recent decades (Tuvendal and Elmqvist 2011). Hence, as good quality water is an important resource for people living in the catchment, and as bird populations in the wetlands in the lower parts of the catchment are declining (H. Cronert, and S.-E. Magnusson, pers. comm.), it is vital to mitigate this negative trend.

A starting point to understand how the catchment is influenced by forest landscape use is to map land owners and actors and their use of different kinds of goods and benefits including the full range of forest landscape values. We used Merlo and Croitoru’s (2005) approach to classify benefits in terms of forest goods, services, and values into use and non-use values, but without applying their total economic value approach to estimate market values, markets for substitute products and potential market values. We rated the interviewees’ answers, and value profiles are presented for each of four main forest owner categories. Finally, we discuss the challenge of communicating different economic values among stakeholders, a broader suite of forest management systems, and the need for fora for collaboration and spatial planning.

## Methodology

### Helge å Catchment as a Case Study

This study focuses on the entire River Helge å catchment, which covers 4725 km^2^. The river runs from the southernmost boreal forest in Sweden (Sjörs 1967), passing temperate lowland temperate deciduous and Scots Pine (*Pinus sylvestris*) sandy forests to the Baltic Sea. While the average forest cover in the catchment is 64 % (Electronic Supplementary Material, Table S1), there is a clear gradient of decreasing forest land cover from upstream north to the downstream south (Electronic Supplementary Material, Fig. S1). The outer border of all municipalities located within the River Helge å catchment including a 5-km buffer zone was used to delimit the total study area (Electronic Supplementary Material, Fig. S2). The study area thus covers 11 336 km^2^ and encompassed the territory of 14 different municipalities in two historical provinces (landskap in Swedish) once belonging to Denmark and Sweden, respectively, and three county administrative regions.

### Mapping Land Owners

Ten groups of land owners were identified based on the analyses of the coarse land ownership maps (Electronic Supplementary Material, Table S2). The ownership landscape was dominated by non-industrial private forest (NIPF) owners who were in possession of 88.6 % of the land. The three other main forest owner categories were Sveaskog Co. (3.3 %), municipalities (1.8 %), Church of Sweden (1.6 %), and the Swedish Environmental Protection Agency (0.8 %). Forest owner groups owning less than 0.5 % of the land were considered to be of minor importance and were excluded from further investigation.

To identify the value profiles of the forest owner groups, telephone interviews were conducted with a sample of interviewees representing the land owner categories. Of a total number of 105 randomly chosen persons contacted by telephone 89 persons were interviewed in 2007. Seventeen NIPF owners were excluded for various reasons (e.g., recent shifts in property ownership, not found, or unwilling to participate). Thus the response rate of NIPF owners was 77 %. This sample consisted of 58 NIPF owners, 25 municipal representatives, 3 County Administration Board (CAB) representatives, 1 executive of Sveaskog Co. management unit in southern Sweden, and 2 managers responsible for the forest management of the Church of Sweden.

The NIPF owners were between 35 and 83 years old, the average age was 58. Of these, 14 were women (24 %) and 44 were men (76 %). NIPF owners and municipalities were both divided into two groups based on the historical provincial units Skåne (downstream) and Småland (upstream). Sveaskog Co. and the Church of Sweden were assigned to the same group as both of them had similar objectives in terms of a focus on wood production. To select NIPF interviewees all forest properties with a forest cover between 19 and 100 ha belonging to NIPF owners were identified. This represents the average size of a forest property in Southern Sweden (N.-G. Cato, pers. comm.). A total of 75 forest properties, evenly distributed between the Forest Agency’s three districts (two in Skåne and one in Småland) within the study area, were selected randomly and the owners were asked to participate in a telephone interview. For all 14 municipalities, the responsible officer for forest management at the municipality was contacted. In most cases, a second person responsible for environmental issues was also contacted for supplementary comments about nature conservation strategies and recreation. The state-owned land set aside for conservation and recreation is owned by the Swedish Environmental Protection Agency (SEPA). However, interviews were conducted with staff at the three County Administration Boards (CAB) within the study area (i.e., the counties of Skåne, Kronoberg and Jönköping), who manage the protected areas and were thus assumed to possess deeper knowledge about local conditions. The CABs’ main responsibility is to coordinate the development of the county in line with goals set in national policy, and is in most cases responsible for the operational management of the state-owned land. In addition the executive at Sveaskog Co. and two managers in charge of the forest management at each of the Church of Sweden’s two dioceses were interviewed.

All telephone interviews were semi-structured (Kvale and Brinkman 2008) and based on four sustainability themes: (1) economic focus, (2) social activities, (3) biodiversity and nature conservation, and (4) historical/cultural aspects. A semi-structured interview is a flexible interview method that allows for new questions to be brought up during the conversations depending on the responses from the interviewees. An interview manual framework was developed based on the total economic value approach. The telephone interviews were recorded digitally and summarized briefly afterwards, but were not transcribed word by word.

### Use and Non-use Values

The Total Economic Value (TEV) concept intends to cover the full range of multiple values of forest ecosystems through estimation of use and non-use (or existence) values (Krutilla 1967; Jacobsson and Dragun 1996) (Table 1). A coherent analytical framework is needed to ensure that these benefits are considered systematically and comprehensively, but without double counting (Pak et al. 2010; de Groot et al. 2010). For analytical reasons TEV of forest can be decomposed into either more general components, or be split up into more detailed ones. Merlo and Croitoru (2005) employed an analytical framework based upon the TEV concept. This straightforwardly classifies real and potential benefits into direct and indirect use values, option values and non-use values. Direct use values include (1) consumptive (e.g., wood and non-wood forest products) and other provisioning services of forest ecosystems in terms of Millennium Ecosystem Assessment classification (MEA 2005) as well as (2) non-consumptive direct use values in terms of landscape quality or recreation (cultural forest ecosystem services). Indirect use values include ecosystem services supplied by watersheds, such as water purification and carbon sequestration, flood regulation, soil formation, and other regulating and supporting forest ecosystem services. To simplify the study and to avoid dealing with uncertainty, option values were assigned to belong to the direct use values and were not treated as an individual value category. Non-use values are not linked to the actual use of forests but rather to conservation interests of the landscape. Two examples are (1) bequest values arising from placing a value on the conservation of natural or cultural elements of the landscape for the benefit of future generations, and (2) existence values derived from the knowledge of conserved ecosystems, habitats, or species. In practice the only difference between existence and bequest values is whether or not the consumer enjoys the existence of a good or service exclusively, or as a potential source of utility for next generations as well.

**Table 1:** Use and non-use value variables recognized by stakeholders and grouped in different value categories and assigned to the four aspects of SFM

Sustainable forest management criteria	Use values				Non-use values	
	Direct use values			Indirect use values	Bequest values	Existence values
	Consumptive		Non-consumptive			
	Wood products	Non-wood products				
Economic	Wood production Fuel wood	Berries Mushrooms Hunting Investment	Landscape quality		Inheritance	
Ecological	Wood production			Soil protection Water protection		Biodiversity Habitat conservation
Social	Wood production	Berries Mushrooms Hunting	Recreation Landscape quality Cultural elements Biodiversity	Soil protection	Cultural elements Habitat conservation	
Cultural	Wood production		Landscape quality Cultural elements		Inheritance Cultural elements Habitat conservation	

The importance of various value variables were rated on a three-graded scale ranging from 0 to 2, where the rank numbers represent the interviewer’s perception of the interviewee’s interest in a particular kind of forest use as being unimportant (0), of lesser importance (1) and of greater importance (2), respectively. The individual value variables used in this survey were selected in an attempt to encompass all kinds of values of the forest landscapes in the study area, and to correspond as closely as possible to the theoretical framework of Merlo and Croitoru (2005), but without moving into economic valuation. The value variables investigated were wood production, fuel wood, berries, mushrooms, hunting, investment, recreation, landscape quality, soil protection, water protection, inheritance, cultural elements, habitat conservation, and biodiversity. However, there are several additional value variables mentioned as part of the TEV concept that have not been considered in this survey (e.g., forest grazing, carbon sequestration, educational, or scientific values). The evaluation was made by the interviewer and was based first of all on the stated opinions of interviewee, but also checked for consistency based on his/her description of how the forest was actually managed.

The value variables were grouped in different value categories and the sums of the rank numbers of every value category were calculated for each forest owner group. In order to visualize the comparison between the different owner groups, an index based on a ratio scale from 0 to 100 % was calculated as the sums of value categories divided by the maximum possible score. The ratio index value was never based on a total sum of less than 12. The resulting value profiles of each of the four main user groups are presented both as bar charts for all value variables, and as radar diagrams following Bossel (2001).

## Results

### Use and Non-use Values

#### Direct Use Values: Consumptive Values

Consumptive timber forest products were reported as one of the major direct use values, which provided land owners with an economic income from production of timber, pulpwood, wood chips, or biomass. The interviewees also mentioned other values connected to forest management. The opinion that silviculture is important to create habitats for biodiversity was expressed among the interviewees. Silvicultural practices such as planting and pre-commercial thinning also served as a social purpose, for example, as a recreational activity or as an emotional enjoyment of creating something. Silviculture was also perceived as an integrated activity of maintaining the cultural landscape.

Non-wood products were recognized by interviewees as values derived from forests. The non-wood values were related to economic as well as social and cultural dimensions. One example was hunting, which was reported to generate meat to the land owner or income in form of leasing the right for hunting. Hunting also represented a popular social event and part of the rural culture. A similar type of value was associated with collecting berries and mushrooms. While this did not generate any important economic income, it was reported as an important recreational value. Other consumptive non-wood values had a strict economical focus. Exploitation of forestland for establishment of residential or industrial areas by municipalities was one example. Some land owners also saw forest ownership as an economic investment, for example, because of the expected increase in value of forest properties.

#### Direct Use Values: Non-consumptive Values

The non-consumptive direct use values included a wide range of values such as landscape quality, recreation, cultural elements, and biodiversity. All these values were connected to social aspects in some way. In addition, the cultural elements and the landscape quality were part of the inhabitants’ cultural identity and sense of place. Some land owners also reflected upon the economic aspect of the landscape quality in terms of increasing the attractiveness and, consequently, the market value of the property by improving the aesthetical qualities. On a regional level, landscape quality often played an important role for the municipalities, since it may improve the attractiveness of the region and thus for immigration and thus improved municipal economy.

#### Indirect Use Values

With the exception of soil and water protection, few indirect values were identified by the interviewees. Some of the interviewees expressed a wish to care for streams by leaving buffer zones and others expressed an ambition to minimize the soil damage during harvesting operations. These values were clearly connected to the ecological dimension of sustainable forest management, but also in some way to the social dimension, for example, in terms of a desire to avoid reducing the quality of important recreation areas.

#### Non-use Bequest Values

Several forest owners considered conservation of cultural elements or particular forest habitats to be important. This ambition included both a wish for future generations to be able to experience these landscape components, and also in a wish to conserve the cultural tradition. Inheritance was another bequest value recognized by forest owners. This value was also linked to the cultural and sense of place contexts, but there was also an economic dimension to it, i.e., a wish to contribute to the financial situation of interviewees’ heirs.

#### Non-use Existence Values

Interviewees also recognized the existence values of the forest landscape, which were linked to the intrinsic values of biodiversity conservation and specific forest and woodland habitats.

### Owner Groups’ Value Profiles

#### Non-industrial Private Owners

**Fig. 1:**
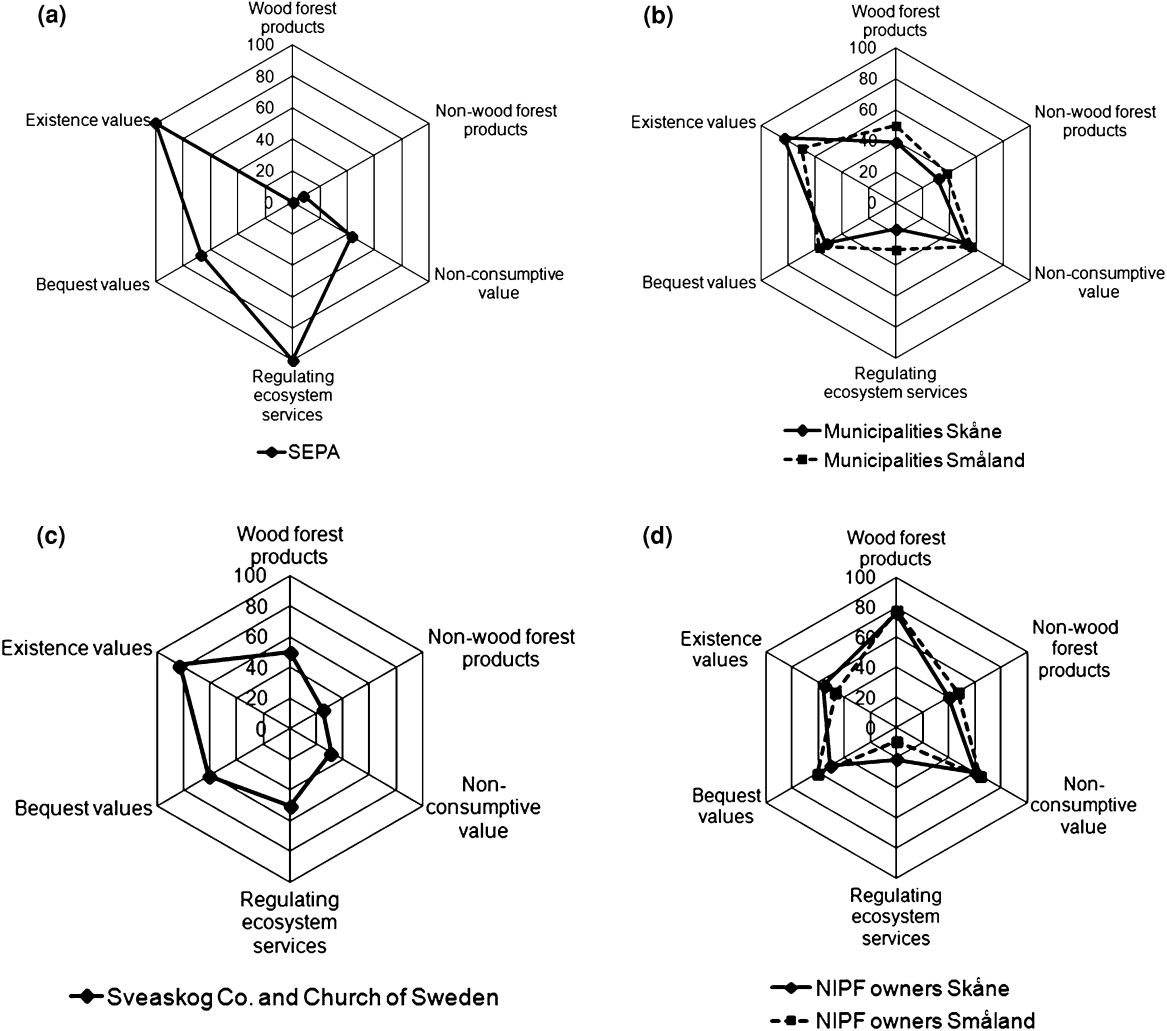
Radar diagrams showing the profiles of **a** NIPF owners in Skåne and Småland, **b** municipalities in Skåne and Småland, **c** Sveaskog and the Church of Sweden, and **d** the land owned by the Swedish Environmental Protection Agency. Index scores based on a scale from 0 to 100 calculated as the sums of value categories divided by the maximum possible score (number of interviewees times 2)

**Fig. 2:**
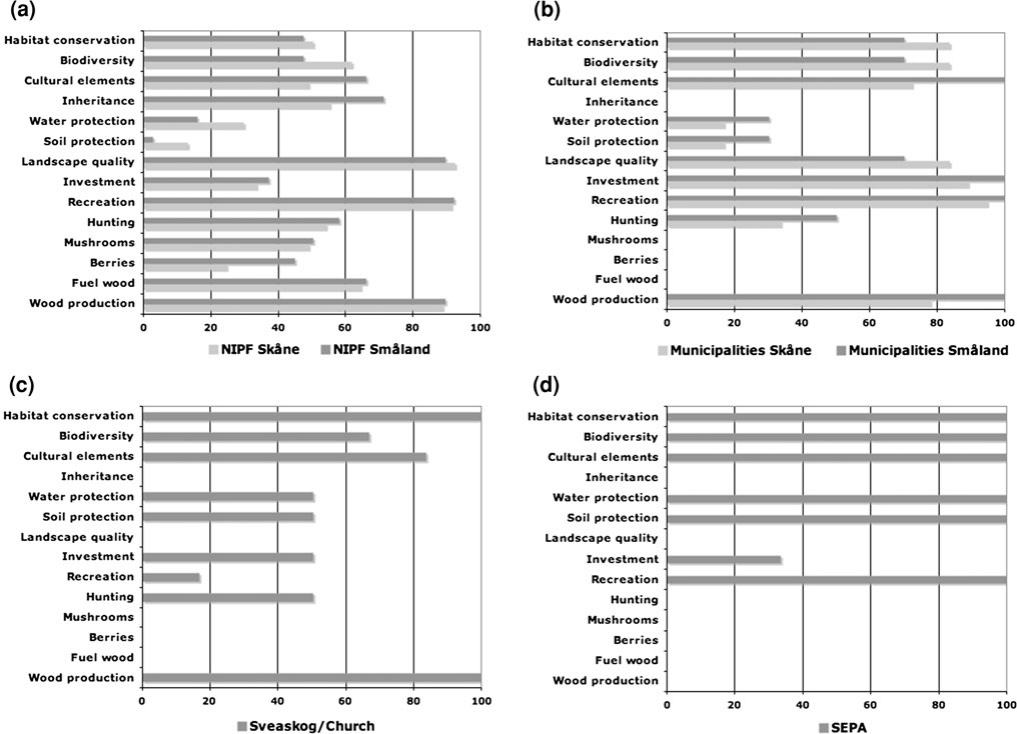
Profiles of use of forest landscape goods, services and values among **a** NIPF owners in the historical provinces Skåne and Småland, **b** municipalities in Skåne and Småland, **c** Sveaskog and the Church of Sweden, and **d** the land owned by the Swedish Environmental Protection Agency

The NIPF owner group was heterogeneous with a diverse profile of use of their forest ownership. What mainly distinguished this owner group from the others is the strong personal and emotional connection to the forest property. While some forest owners explicitly stated that the purpose of their forest ownership was to generate income, the majority seemed to have a more complex approach where the economic factor played a minor or complementary role. The value profiles (Fig. 1a) point to important consumptive wood and non-wood values as well as non-consumptive, bequest, and existence values. The regulating ecosystem services values soil and water protection were, however, perceived as relatively unimportant. The most important use values to non-industrial private owners were wood production, recreation, and landscape quality (Fig. 2a).

#### Municipalities

The value profiles of the municipalities indicated high importance for option, bequest, and existence values as well as some direct use values, whereas indirect use values and some direct use values seemed to be of lesser importance. The direct use values were primarily connected to recreation, timber production, and landscape quality. Most municipalities used forested land for production purposes with different degrees of intensity. While some municipalities had determined a revenue target from forest management, other municipalities seemed to have different priorities.

The municipalities’ value profiles indicate that all value categories except the regulating ecosystem services values were perceived as important (Fig. 1b). The use values perceived as most important for the municipality owner groups were wood production, recreation, investment, landscape quality, cultural elements, biodiversity, and habitat conservation (Fig. 2b). The importance of hunting, mushrooms, and berries were perceived as less important, and soil and water protection as fairly unimportant.

#### Forest Industry

The primary objective of the Church of Sweden’s forest ownership was to earn money from forest management. Sveaskog Co. shared this goal but had explicit other important objectives as well. The value profile for these two owner types showed that existence values and bequest values were ranked high while indirect use values were rather low. In order to generate income, the main focus of the forest management was production of biomass. The largest part of the income was derived from timber and pulpwood production. In addition, biomass residues from timber harvesting are turned into wood chips that are sold as bioenergy fuel. The leasing of hunting rights also generated some profit. Non-timber forest products were of no direct interest for these two forest industrial owners. The recreational values were of some importance to Sveaskog Co., which recently has founded a subsidiary company (Sveaskog Naturturism AB) that deals primarily with wildlife tourism. Concerning the indirect use values the interviewees pointed out that the necessary precautions and considerations close to watercourses or regarding soil protection are stipulated by the national legislation. Investment in forest properties was of little importance but all three interviewees agree that it may sometimes be advantageous to trade a forest property for another to improve management efficiency, for example, related to logistics. The importance to conserve the cultural heritage of the region (bequest values) was acknowledged by all three interviewees. Once again, the requirements stipulated by the legislation were being respected and the representatives of the Church of Sweden pointed out that they have decided to indicate valuable cultural remains with signs in the field. Conservation of biodiversity (existence values) was claimed to be very important by all three interviewees. All forests are certified according to the FSC system and the Church of Sweden’s land was in addition certified under the PEFC system. Conservation of biodiversity represented one of the core management objectives of Sveaskog Co., and for the Church of Sweden it is important to appear as a responsible forest manager. Conservation of habitats was first and foremost related to conservation of biodiversity. All three interviewees stated that they have set aside more land for nature conservation purposes compared to what is necessary to fulfill the minimum requirements of the forest and environmental policies and the certification standards.

The value profiles indicate that the value categories of importance were the consumptive wood, existence, and bequest values (Fig. 1c). To Sveaskog Co. and the Church of Sweden, the use categories perceived as most important were wood production, habitat conservation, and cultural elements (Fig. 2c). The importance of biodiversity was also high. Soil and water protection, energy wood, investment, and hunting received intermediate scores and the importance of the remaining value variables were low.

#### Swedish Environmental Protection Agency

The value profile of the SEPA was clearly different from the other owner group profiles. The objective of forest ownership by the SEPA is focused solely on conservation interests. Existence and bequest values were therefore the most important ones. As a consequence, indirect use values (soil and water protection) were also highly ranked. Another major objective was to promote and encourage recreational activities. Occasionally, this objective could be stronger than the focus on conservation of species. Besides recreation, no other direct use values were recognized. However, two of the county administrative boards also acquired land to be used to compensate private land owners when nature reserves were established on their land.

The value profile indicates that only existence and regulating ecosystem services values were highly important (Fig. 1d). Bequest values and non-consumptive values displayed intermediate scores, and the consumptive wood and non-wood values were very low. The SEPA owner group (Fig. 2d) rated the value variables recreation, cultural elements, biodiversity, habitat conservation, as well as soil and water protection to be most important. Investment was of some importance while the remaining categories were of no importance.

## Discussion

### The Challenge of Economic Valuation

A wide range of international and national policies related to the ecologically, economically, and socio-culturally sustainable use of renewable natural resources have been formulated globally since the appearance of the sustainability discourse during the late 1980s (Kennedy et al. 2001; Campbell and Sayer 2003; Innes and Hoen 2005; Saastamoinen 2005). Three European examples are the Pan-European forest policy process (MCPFE 1993), the European Landscape Convention (ELC 2000), and the EC Water Framework Directive (WFD 2000). Implementing such ambitions requires that landscapes’ stakeholders and actors have comprehensive information about what is produced in the landscape and its consequences for other stakeholders and values (Norgaard 2010). This transparency is also a prerequisite for effective collaboration among different societal sectors and levels of governance at multiple levels (Borrini-Feyerabend et al. 2004; Adger and Jordan 2009; Elbakidze et al. 2010). As Lee (1993) pointed out, there is a need for both a compass that shows the state and trends of sustainability as pronounced as desirable in different policies, and a gyroscope in terms of participation in governance processes.

The interviewees’ answers showed that there was a broad range of use and non-use values in the River Helge å catchment, and no difference between upstream and downstream profiles. The direct use values were very important for all groups, but the types of values varied. In this aspect, the SEPA forest ownership had a clear focus solely on conservation and recreational values. By contrast, Sveaskog Co. and the Church of Sweden had a profile that concentrated primarily on wood and biomass production for the region’s forest industry and bioenergy consumption, respectively, while the municipalities had a more diverse value profile. These results are consistent with the mandates of these forest owner categories. The fact that both existence and bequest values were still perceived as important for Sveaskog Co. and the Church of Sweden reflect these companies’ policy towards forestry. The NIPF owner group showed the most diverse value profile, including also non-consumptive use, indicating that many private land owners see many values in their forests that go beyond the production of wood. This has been observed also in other studies (e.g., Stenseke 2006). It can be argued that emotional values correspond to what Merlo and Croitoru (2005) refer to as sensibility values, identity values, and aesthetical values, which are not quantifiable in monetary terms. At a more general level, the diverse profile among forest owner categories is consistent with the high importance associated to multiple benefits of forests as pronounced in current policies (e.g., Boman et al. 2000; Kindstrand et al. 2008).

Since it is difficult to identify true non-use values of forest owners, categorization of biodiversity and intact ecosystems as ‘non-use’ value is conditional. However, it is clear that forest owners who concentrated on biodiversity conservation and thus received significant ‘non-use value’, provide a source of true non-use value for those stakeholders who derive positive utility from the very existence of high level of forest biodiversity in the River Helge å catchment.

Investigations concerning stakeholders’ perceptions of forest ecosystem services, which are methodologically similar to that presented in this study, have been conducted for North-western Ontario (Kant and Lee 2004) and Western Ukraine (Zahvoyska 2008). These studies applied the Conceptual Content Cognitive Mapping method (Kearney and Kaplan 1997), a tool for non-market oriented stated preference to identify stakeholders’ values associated with forests, and non-parametric statistics to develop statistically significant sets of cognitive maps of stakeholders’ preferences associated with forests. For instance the suite of forest values identified in the Ukrainian case study consisted of nine dominant themes and 37 sub-themes. Four stakeholder groups were investigated: Local population, Forest industry, Environmental non-governmental organizations, and City population. The set of cognitive maps of stakeholders’ preferences, regarding forest values, indicated that Environmental, Recreational, and Economic values were the most appreciated ones by respondents. Tourist values and Health care received the smallest attention from the respondents. From the stakeholders perspective the forest values universe shows that Local population verbalized the widest palette of values. Derived cognitive maps prove statistically significant differences in forest values profiles across stakeholders (Zahvoyska and Bas 2009). Similarly, this study about the River Helge å catchment supports the hypothesis by Boyd and Banzaf (2006) that people are more familiar with ecosystem services which affect their wellbeing in direct ways [like provisioning, regulating and cultural services, i.e., the MEA (2005) classification] and are ignorant about supporting services like primary productivity, biogeochemistry.

It is important to point out that the method of semi-structured interviews used in this study does not demonstrate trade-offs between the different value components in actual or hypothetical choice situation, and therefore cannot be used for estimation of the concerned values in monetary terms explicitly. However, the simple ranking approach can approximate forest-owners’ value profiles. The quantitative visualization based on numerical analyses of rank values represents a major simplification of the reality. In future studies it could be advisable to let the interviewees rate their own opinion themselves. What are the subjective views of different forest owner categories, and other stakeholders? What are the ideologies or cultures among stakeholders? How do they match this with their practices? Moreover, it is necessary to point out that economic value rather reflects the lower bound of the true value of the full suite of forest benefits including environmental goods. Intrinsic, spiritual, patriotic and other types of value that people use to assign to the forest are not necessarily included into economic analyses since economic valuation techniques often fail to account for these and similar components of value (Costanza et al. 1997).

### Forest Management, Planning, Collaboration, and Education

Mapping perceptions and values of different land owners concerning ecosystem services represents an important step towards developing adaptive landscape management (Lindström et al. 2006; Tikkanen et al. 2006; Nordlund and Westin 2011). The broad use profiles in this study stresses the need for new forest management regimes (Axelsson and Angelstam 2011), forest planning businesses that provide broader management advice (Uliczka et al. 2004), improved communication and learning among stakeholders (Axelsson et al. 2013b), and broadened content in forestry educations (Axelsson and Angelstam 2011).

The traditional industrial forest management system based on clear-felling with tree retention is currently contested by actors who advocate uneven-aged or cohort management systems for both ecological and socio-cultural reasons (Axelsson et al. 2007; Siiskonen 2007; Tahvonen 2009). This applies to urban forests, and forest owners that own forest for other than monetary reasons (Kindstrand et al. 2008). There is also increased interest in viewing forest and woodland landscapes’ natural and cultural capital as an infrastructure for tourism and recreation (Vail and Hultkrantz 2000).

While the large number of forest owners is a challenge for landscape planning in terms of large spatial extents (Sandström et al. 2011), the multifaceted profiles of forest owners in this study provides opportunity for new planning approaches with a focus on a broader range of landscape values than wood.

The clear difference among the forest owner categories’ value profiles shown in this study is a challenge for collaboration. The emergence of ecosystem services derived from waters, which are affected by the surrounding forests (e.g., Tuvendal and Elmqvist 2011), is likely to reinforce this. While multi-level learning takes time (Axelsson et al. 2013b), this is necessary to accommodate different interests. Empowering communities and municipalities is one approach.

Higher education of professionals is a critically important way of supporting the implementation of sustainable forest management policy. In line with the contemporary forest industrial regime, forest management education in Sweden is oriented towards the production of wood (Bergqvist et al. 1989). However, new forest policies may result both in large changes in forest management education (Hosny El-Lakany 2004) and inertia (Siiskonen 2010). This stresses the need to equip students with interdisciplinary and transdisciplinary skills (Fry 2001; Hammer and Söderqvist 2001; Axelsson 2010).

### Governing Forest Landscape Values

The Kristianstad Vattenrike Biosphere Reserve (KVBR) within the River Helge å catchment has been subject to several studies describing the social and organizational processes behind the development of landscape governance. In particular, the role of agreed perceptions and visions, stakeholder coordination and networks, institutional arrangements, and individual leadership have been emphasized (Olsson et al. 2004, Hahn et al. 2006; Olsson 2007; Schultz et al. 2007; Hahn 2011). While these studies have provided important knowledge on some aspects of the sustainable development process, and the emergence of an adaptive social–ecological system within KVBR, other dimensions have received much less attention. In the Millennium Ecosystem Assessment, KVBR was brought up globally as a good local example of sustainable development (MEA 2005). While research so far has concentrated on the adaptive governance of the biosphere reserve no one has yet done an integrated assessment of the areas’ different sustainability dimensions, i.e., assessed the outcome on the ground of the adaptive governance process. For instance, very few studies have been made on the functioning and resilience of the major ecosystems within the area. Also, most studies have not taken a landscape approach (see Axelsson et al. 2011) at the catchment level but have been restricted to more limited activities and areas within the biosphere reserve, and have focused on the southernmost part of the river catchment. An exception is a recent study by Tuvendal and Elmqvist (2011) who analyzed the effects of brownification of the River Helge å, originating in the forested upper parts of the catchment, on ecosystem services in downstream areas, and how stakeholders responded to these effects. Such catchment-level approaches are badly needed and provide more ecological realism, since widely different ecosystems within a catchment are often connected and influence each other. We emphasize the need for studies that assess the present status and development trends, the adaptive governance process and its outcomes on the ground, including studies that take an integrated approach trying to assess all dimensions of sustainability (see Lee 1993; Rauschmayer et al. 2009; Angelstam et al. 2013). This also means that values and attitudes of stakeholders and actors in different parts of the catchment must be understood. By focusing on forest ecosystems and the services provided to forest owners within the River Helge å catchment, our analysis is a first step to understand how management and governance of forest landscapes could match the forest owner’s value profiles. We see this is as an important complement to previous studies on the opportunities for adaptive governance emerging in the KVBR. Ideally, given the upstream–downstream links in this catchment (Tuvendal and Elmqvist 2011) the KVBR work should be extended from the focus on the downstream part of the catchment towards the development of a landscape approach for the whole watershed.

## Conclusions

This study shows that different forest owner categories have different profiles of use and non-use forest values. The attainment of the current policy visions of forest, environmental, rural development, and others advocating sustainability in landscapes with heterogeneous forest owner composition, requires collaboration among stakeholders. The participants in this collaborative learning process need to develop an understanding of current policies regarding landscapes, communicate their own goals, values, and perceptions, and understand how this affects other interests. Another key issue is to be aware of the status and development trends of all sustainability dimensions (Andersson et al. 2012). Municipal comprehensive planning, Biosphere Reserve (Price 2002), Model Forest and EU Leader (Ray 2000) are examples of approaches that aim at supporting informed multi-level governance toward sustainability (Sayer and Maginnis 2005). However, land ownership rights are strong in Sweden (e.g., Sandström et al. 2011), which stresses the role of solutions that are adapted to the contexts of different forest owner categories.

## Electronic supplementary material

Below is the link to the electronic supplementary material.

Supplementary material 1 (PDF 147 kb)
