# Supplementary material for: Multifaceted Value Profiles of Forest Owner Categories in South Sweden: The River Helge å Catchment as a Case Study
Source: Ambio. 2013 Mar 10;42(2):188–200. doi: 10.1007/s13280-012-0374-2 (PMC3593028; doi:10.1007/s13280-012-0374-2)
Supplement: Supplementary file 1 — Supplementary material 1 (PDF 147 kb) [file 13280_2012_374_MOESM1_ESM.pdf]

Electronic Supplementary Material

Multi-faceted value profiles of forest owner categories in South Sweden: the River Helge å catchment as a case study

Gustav Richnau, Per Angelstam, Sviataslau Valasiuk, Lyudmyla Zahvoyska, Robert Axelsson, Marine Elbakidze, Joshua Farley, Ingemar Jönsson, Ihor Soloviy

Table S1 Land cover proportions in the Helge å river catchment study area

Land cover category	Area (ha)	Proportion (%)
Urban area	21 831	1.9
Forest	730 128	64.8
Open land	248 207	21.9
Wetland	44 532	3.9
Open water	83 897	7.4
Total	1 133 594	100

Table S2 Land owner categories with more than 1000 ha in the River Helge å catchment study area (11 336 km2)

Land owner category	Inter-views	Land cover area (ha)	Area proportion of study area (%)
Other landowners	No*	16 334	1.4
Incorporate companies	No*	18 934	1.7
Bergvik Skog AB	No*	1 897	<0.5
The National Fortifications Administration	No*	5 188	<0.5
The National Property Board	No*	2 195	<0.5
Municipalities	Yes	20 433	1.8
Church of Sweden	Yes	17 714	1.6
Swedish Environmental Protection Agency	Yes	8 860	0.8
Sveaskog Co.	Yes	37 482	3.3
Non-industrial private forest owners	Yes	1 006 789	88.6
Sum		1 133 929	100

* Land owner groups owning less than 0.5 % were excluded from further interviews. The opinions of incorporate companies and other landowners were assumed to be represented by the remaining owner categories and were also excluded.

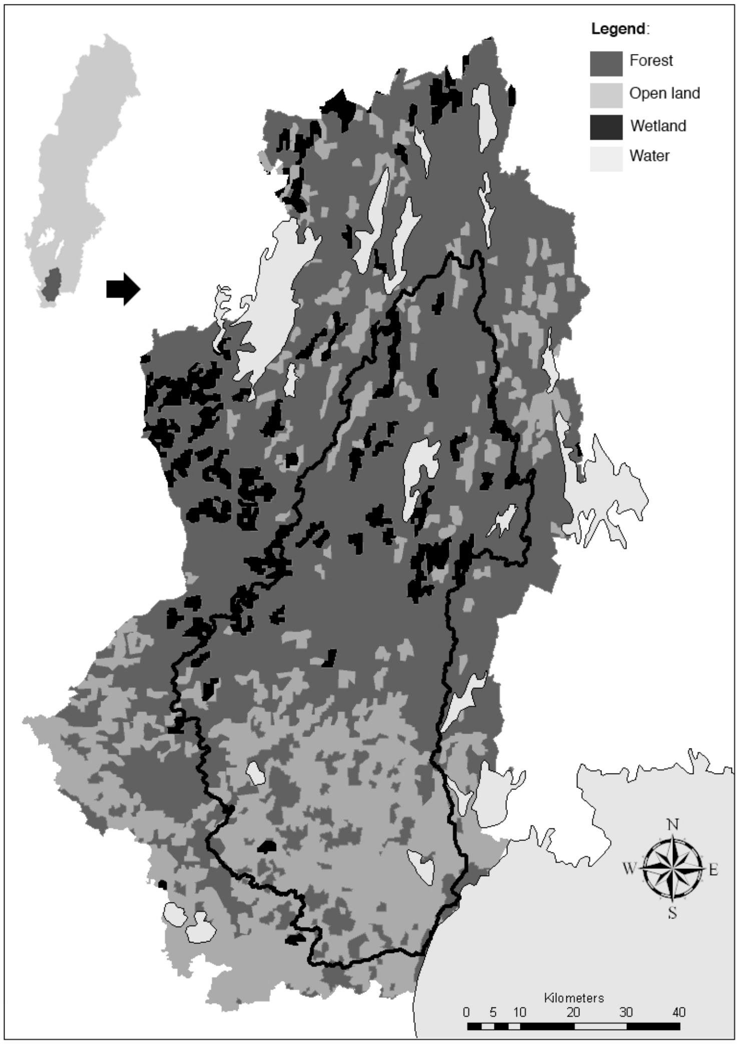

Fig. S1 Map showing the main land cover types of the study area and the River Helge å catchment

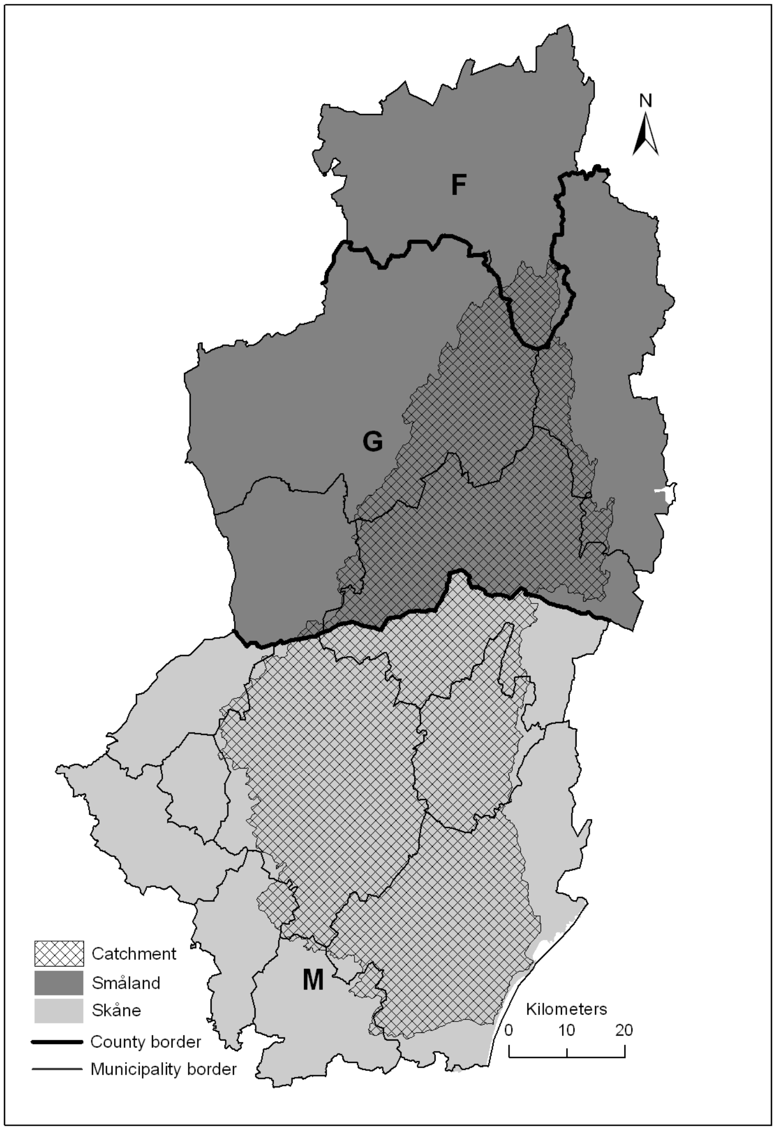

Fig. S2 Map showing the 14 municipalities, the three county administrative boards (F=Jönköping county, G=Kronoberg county, M=Skåne county) and the two historical provinces (Skåne and Småland) within the study area and the River Helge å catchment
